# Electron beam melting in the fabrication of three-dimensional mesh titanium mandibular prosthesis scaffold

**DOI:** 10.1038/s41598-017-15564-6

**Published:** 2018-01-15

**Authors:** Rongzeng Yan, Danmei Luo, Haitao Huang, Runxin Li, Niu Yu, Changkui Liu, Min Hu, Qiguo Rong

**Affiliations:** 1 Department of Stomatology, Chinese PLA General Hospital, Beijing, 100871 China; 2 Department of Mechanics and Engineering science, College of Engineering, Peking University, Beijing, 100871 China; 3 Oral and Maxillofacial Surgery department of General Hospital of Liao he Oilfield, Panjin, 124000 Liaoning China

## Abstract

The study was designed to fulfill effective work-flow to fabricate three-dimensional mesh titanium scaffold for mandibular reconstruction. The 3D titanium mesh scaffold was designed based on a volunteer with whole mandible defect. (1) acquisition of the CT data; (2) design with computer aided design (CAD) and finite element analysis (FEA). The pore size and intervals with the best mechanic strength was also calculated using FEA. (3) fabrication of the scaffold using electron beam melting (EBM); (4) implantation surgery. The case recovered well, without loosening and rejection. Additionally, 12 mandibular defect model beagles were used to verify the results. The model was established via tooth extraction and mandibular resection surgeries, and the scaffold was designed individually based on CT data obtained at 2 weeks after extraction operation. Then scaffolds were fabricated using 3D EBM, and the implantation surgery was performed at 2 months after extraction operation. All the animals healed well after implantation, and the grafted mandibular recovered well with time. The relevant parameters of the grafted mandibular were nearly to the native mandibular at postoperative 12 months. It is feasible to fabricate mesh titanium scaffold for repairing mandibular defects individually using reverse engineering, CAD and EBM techniques.

## Introduction

Reconstruction of mandibular defect should restore the anatomical height and contour of the missing part, meanwhile optimal restoration of function involves mastication, deglutition and the management of oral secretions^1–3^. Surgeons have been trying to reconstruct mandibles for more than a century. Using autologous bone grafting, especially a vascularized fibula free flap transfer is a standard surgical procedure representing major treatment of mandibular reconstruction^4^. Autologous grafts have a number of limitations, for instance, limited availability and donor site morbidity, including residual pain, serious blood loss and the complicated operative technique^5^. However, with the development of biotechnology, tissue engineering might provide a new clue for the evolution of mandibular reconstruction. The restoration of bone tissue defects perhaps have a great potential to enhance the feasibility of bone regeneration^6,7^. In recent years, bone substitutes made by titanium exhibit good mechanical strength and biological compatibility which have been widely used as biomedical materials to replace dysfunctional hard tissue in human body. There is a trend in orthopedics implants towards personalized metal implants, including porous parts added to dense core implants and also porous “biometals”, to repair bone defects, which is being used more and more in the clinic^8^. None of the present available techniques can meet all these needs, so the efforts for a better means of reconstruction should continue to make. With the development of the combinations of computer technology and medical science, more and more methods are used to reconstruct mandible. Only a few successful clinical application cases of mandible reconstruction have been published so far, reported by Warnke *et al*.^9^. To solve the too-low mechanical strength in early period of implanting loaded tissue engineering bones, our research group proposed a hypothesis of individualized functional repair of mandibular defects using a 3D porous internal tissue engineering titanium scaffold. Three-dimensional mesh titanium scaffold was combined with osteogenic material, chondrogenic material and bone marrow stromal stem cells *in vivo* tissue engineering to repair defects. After bone generation with scaffolds is completed *in vivo*, the patient’s appearance and oral functions will be restored.

The scaffolds require various special functions including improvement of mechanical strength to provide structural support and to guide tissue regeneration, shape recovering of defect tissues. But, it is very difficult and time-consuming to model, analyze and fabricate the whole mesh customized scaffold for all the different range of porosities and pore diameters. Currently, the traditional CAD method has been recommended to design scaffolds. Compared with that using additive manufacturing processes like EBM, patient-specific titanium cellular meshes can be successfully fabricated according to a wide range of designs and modified directly from CAD data, which offers the possibility to create complete formed implants as well as 3D mesh scaffolds with regular arranged structures^10–15^. In comparison with previous methods, it offers the advantages to control internal pore architectures and complex cell shapes accurately, consequently it may be widely used in clinical practices.

The aim of this study was to discuss the rational work-flow for engineering tissues and a 3D mesh internal titanium scaffold by combining 3D reconstruction with EBM technique to repair mandibular defect.

## Modeling and Methodology

A whole mandible defect case from the Radiology Department of PLA Hospital was selected to construct the 3D Titanium modeling of mandibular reconstruction. In addition, 12 beagles were used to establish the mandibular defect animal model to verify the effect of 3D mesh titanium mandibular prosthesis scaffold.

Briefly, 3D reconstruction from CT medical images including the external shape and internal porous structures were designed with commercial CAD software (Unigraphics NX 8.0, EDS) and CAE software (ANSYS 14.0 Swanson Analysis System Co., Houston,TX, USA). The CAD data of the structures was converted into stereolithography (STL) data, which was then imported into Materialise’s Magics software and finally converted into EBM. The samples were produced by an Arcam’s EBM machine (EBM A2 ArcamAB, Sweden). The study procedures were accorded with the Ethic committee of PLA hospital and the Institutional Animal Care and Use Committee. The following methods were carried out in accordance with the approved guidelines. With the approval of Chinese PLA General Hospital Ethics Committee, written informed consent was obtaining from the participants.

### 3D modeling reconstruction based on CT image

#### Medical image acquisition

In this case, the skull of a volunteer with normal occlusion and without temporomandibular joint (TMJ) disease was scanned by spiral CT performed with a GE Healthcare Bright Speed instrument (GE Healthcare, Fairfield, CT) at the Radiology Department of PLA Hospital. It was implemented under the following conditions: 120 kV, 250 mA, 0.625 mm slice thickness, 0.5 mm slice interval, 0.75 s rotation time, and 512 × 512 pixels image resolution. The images of the maxillofacial region acquisition yielded 352 slices, which were recorded on a disc in a DICOM (Digital Imaging and Communications in Medicine) format files.

### Image processing and 3D medical model reconstruction

After the stack of CT slices was yielded, the images were imported and processed with MIMICS (version15.0, Materialise, Leuven,Belgium), which was a medical software of a popular commercial platform. This model was constructed semi automatically by threshold-based segmentation, contour extraction, and surface reconstruction. A 3D digital model of the mandible (without soft tissue) was reconstructed. To construct the triangular model of a bone structure from the volume data, the following steps were performed: threshold of the Hounsfield value setting, growing region and 3D model calculation. To separate the mandible from whole data, the linkage on each image like the TMJ should be erased. The selected bones structure was converted into a 3D STL model. The 3D STL model was used to generate an anatomic model, which served as the basis for CAD modeling of mandible geometry.

### CAD remodeling

The CAD geometry was constructed by reverse engineering in Geomagic. Mandible STL modeling was used as a reference object for the CAD modeling. The reconstructed 3D point cloud bone models were then imported in the commercial package reverse engineering software Geomagic Studio v 12.0 (Research Triangle Park, NC, USA) and to be processed into 3D surface models. Then through the data points for curve reconstruction, surface reconstruction and the external shape of the entity to generate three-dimensional model were reconstructed to create geometric models with non-uniform rational B-spline (NURBS) and then converted into rapid prototyping system for the STL file. Then the triangular model was established, in which linear interpolation was implemented to yield smooth surfaces. The teeth were removed from the model as they made no difference to the mandible.

### Design of the internal microstructure of mandibular scaffold 3D mesh via finite element analysis

Appropriate geometrical structures with specific parameters should be designed and a bio-material with appropriate properties ought to be selected to satisfy the requirements of biological, bio-mechanical, and bio-material functions. In terms of geometrical structure design, two parameters should be composed with porosity and pore size. From previous studies, the porosity has been expected to reach the value ranging from 50% to 90%, and pore size should be from 100 to 500 μm. The two geometrical properties should be kept within an accuracy range of 80–90%, so the mistake could be predicted and compensated in the design phase. The CAD geometric model of the human edentulous mandible was imported into the finite element mesh (FEM). By CAD and finite element analysis (FEA), we established digital model of bone scaffold with optimal mesh structure. In addition, we could also find out the pore size and intervals with the best mechanic strength via FEA. The digital design method was adopted directly based on triangular mesh.

**Figure 1:**
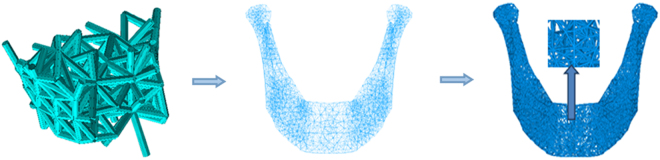
Work-flow of 3D mesh bone scaffold’s digital design three-dimensional finite element mesh of a human mandible.

Firstly, original model was the intact and the edentulous mandible was obtained in STL format. Subsequently, according to the measurement and location of mandibular defects determined by clinical data, a similar area of the dentulous mandible model was regarded as scaffolds design area. When a structure was shaped like the triangle, it would be more strong and stable than other kinds of shapes, so the scaffolds were designed as a series of tetrahedral structures. The complete process flow for CAD/CAM generating implants was shown in Fig. 1. The design area of the model was divided into uniform 3D tetrahedral element mesh type. A FEM with 10-node quadratic tetrahedral elements was built using Ansys 14 (Ansys, Inc, Canonsburg, PA) free meshing. Then, the edges of these tetrahedrons were considered as struts within the scaffold. Finally, the scaffold model was designed and built completely.

### Preparation of Ti6Al4V scaffolds by EBM

In order to verify the validity of the proposed method and manufacturability of the generated scaffold models, a preliminary test of fabrication was conducted. Geomagic Studio was used to import the scan as a CAD model and convert it to the STL format. Three different software tools were used to prepare a job file: Magics, Arcam Build Assembler and EBM Control Simulator. Magics is used for:importing the 3D CAD, drawing and creating an STL representation of the scaffold, modifying and positioning the part in the build envelope, creating supports exporting the scaffold and the support as STL files. The STL format file was inspected using Magics (Materialise; Belgium) for repairing data process. If the STL file was generated without mistakes, it was printed using a 3D printer (Electron Beam Melting A2; Arcam AB; Sweden).

These mesh structures were built layer-by-layer using the Ti-6Al-4V medical-grade powder (Arcam AB; Sweden) with average diameter 80 μm. As shown in Table 1, the chemical composition of the starting powder conformed to the standard for Ti-6Al-4V alloy castings for surgical implants (ASTMF136-98).

**Table 1:** Composition of the used Ti-6Al-4V starting powder in comparison with the standard for Ti-6Al-4V alloy castings for surgical implants (ASTMF136-98).

Element (wt%)	Al	V	C	Fe	O	N	H	Ti
Powder	6.4	4.0	0.02	0.23	0.09	0.01	0.003	BAL
ASTMF136-98	5.5-6.5	3.5–4.5	<0.08	<0.25	<0.13	<0.05	<0.012	BAL

EBM Build Assembler was used for importing the STL files, creating volume supports; slicing STL files into 2D compressed layer files, viewing layer files, assigning as well as assembling layer files into Arcam Build files and exporting Arcam Build files.

The shapes were controlled through the three dimensional CAD using an electron beam melting system for enhancement of melting and productivity. The implant could be placed in a vacuum chamber for low stress on the implant; its performance was superior to implant casting as well as forging, and the beam was able to be close to the implant. Printing was performed at the ambient temperature of 750 ℃, and voltage V = 60 kV, electron beam current 4–8 mA, Layer thickness 0.05 mm.s

### Animal model

In this study, 12 beagles were used to establish the mandibular defect animal model. The mandibular premolar and molar teeth (5 teeth) on the right side were pulled out. The extraction region was more than 50 mm. 2 weeks after teeth extraction operation, the beagles were treated with 3% pentobarbital natrium (1 ml/kg) for anesthesia, and then performed the spiral CT scanning. According to the CT data, a 40 mm block mandibular defect was designed at the right posterior segment of the edentulous area. The 3D mesh titanium mandibular prosthesis scaffolds were conducted based on the related parameters of the animal model. The bone graft stent was supported by the lower part bone surface of the jawbone, and thickened 0.8 mm inward, to form the bracket structure. In the detect area, an extension board with 16 mm in length, 8 mm in width was designed to fix the two ends of defects. Furthermore, the extension board on the buccal surface of mandible thickened 0.8 mm inward. Thus, the extension plate was completely jointed with the mandible bone surface of the corresponding part. A width of 5 mm smooth transition was performed at the bracket-extension plate junction to eliminate sharp steps. Finally, the uniformaly distributed mesh with a diameter of 2 mm was designed on the surface of the bracket, while the diameter of 1.8 mm fixed nail was designed on the extension plate.

Two months after tooth extraction operation, the alveolar mucosa at the extraction region was completely healed, and the mandibular resection was performed for the animals to obtain mandibular defect animal models. Then the prosthesis scaffolds were implanted into the animals. After the implantation, all the animals were received penicillin injection, and liquid diet for 7d.

### Tissues specimens evaluation

Radionuclide bone imaging was performed at 1, 3 6, and 12 months after operation to evaluate the graft. The detailed procedures were according to the previous description^16,17^. The same size in the graft and the opposite side in the native mandible were selected for semi-quantitative analysis. The activity was estimated by the counts per image element on the computer matrix for each region. The ratio of activity between graft and native mandible was used to evaluate the status of graft. The ratio more than 1.03 suggested good recovery, while the ratio less than 0.94 indicated poor recovery.

In addition, 3 animals were executed respectively at postoperative 1, 3, 6 and 12 months. The jaws were harvest and fixed with 4% paraformaldehyde solution. After tray and soft issues removed, each specimen was divided into two parts: one part (about 30 mm) for mechanical test, and the other part (about 10 mm) for micro-CT scan.

Mechanical test was performed with 3-point bending test with a universal testing machine. The parameters were set as follows: the distance between the two support points was 20mmm; the cross-head speed of 5 mm/min until bone failure. The max loads before bone failure were recorded for further analysis. The native mandible from the same animal were employed as controls.

Micro-CT scan: Micro-CT scan was performed for the specimens. The conditions for CT scan were as follows: 44 mltube_21 m_150 min_ss protocol. MicroView ABA 2.1.2 software was used for imaging analysis. The morphological indexes including bone volume fraction (BVF), tissue mineral density (TMD), structure model index (SMI), trabecular number (Tb.N), trabecular thickness (Tb.Tn), trabecular separation (Tb.Sp) were recorded for each specimen. The data of native mandible bone were recorded as control.

### Statistic analyses

All the statistic analyses were performed in SPSS18.0 software (SPSS Inc., Chicago, IL, USA) and GraphPad Prism version 5.0 (GraphPad, San Diego, CA, USA). Each test was repeated in three times, and the data were summarized and shown in mean ± SD. If the data distributions were in accord with normality, parametric student’s t test was applied to evaluate the differences between the two groups, otherwise, non-parametric Wilcoxon rank sum test was used. *P* values less than 0.05 were considered statistically significant.

## Results

### 3D modeling reconstruction based on CT image

**Figure 2:**
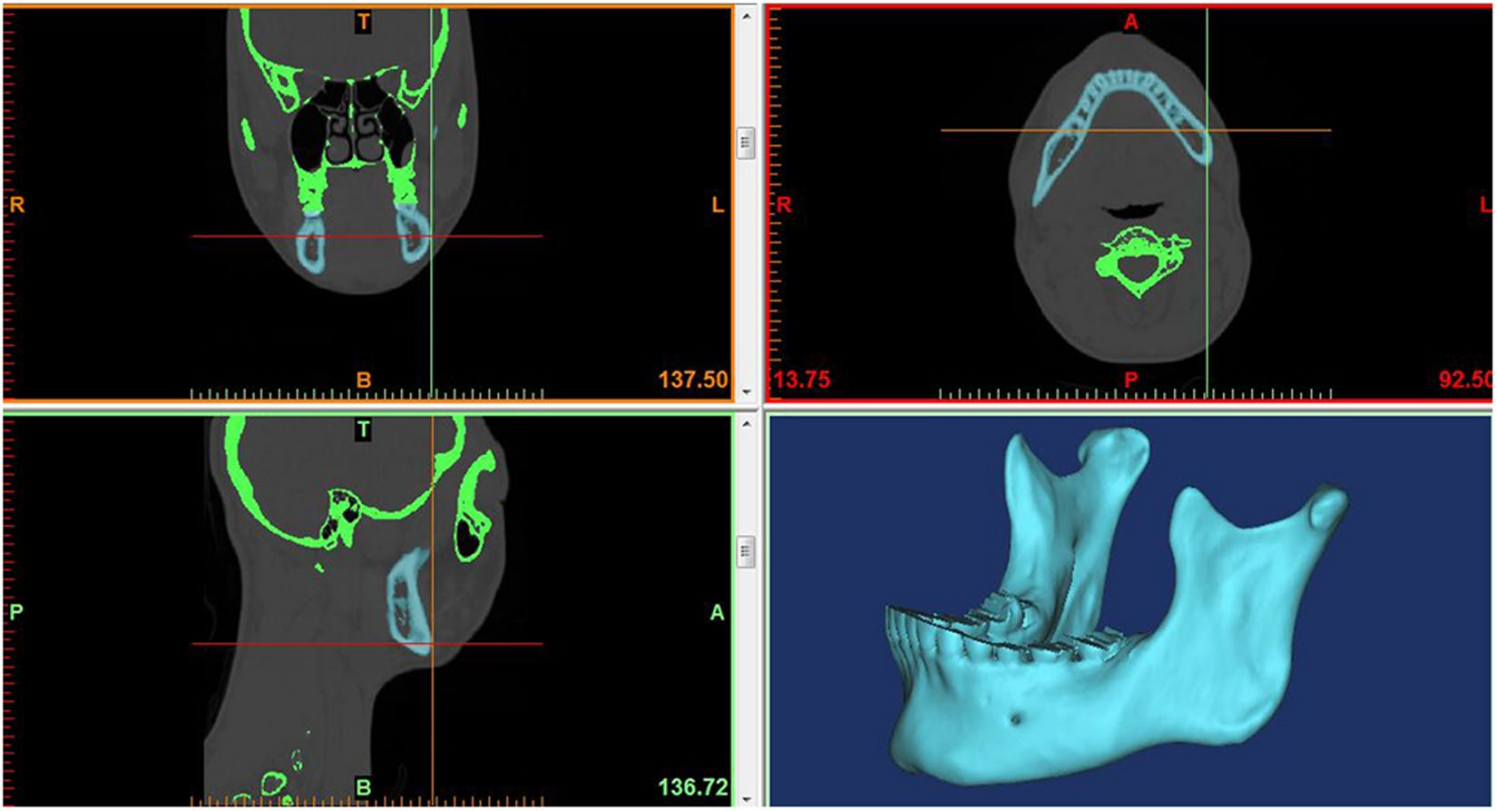
3-D anatomic modeling construction of human mandible.

The main contents of 3D reconstruction from medical images included inputting of medical image, pre-processing, such as filtering and interpolating, segmenting and extracting tissues or organs of body, constructing 3D surface models. As shown in Fig. 2, we got the 3D modeling construction through spiral CT scan.

### 3D surface models was constructed by CAD

**Figure 3:**
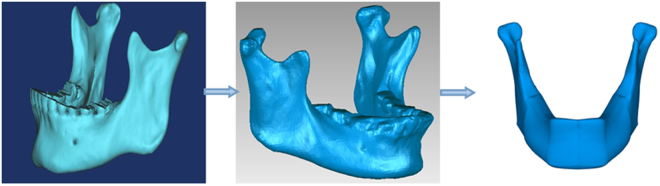
Reconstructive process of CAD model of human mandible.

After we got the 3-D anatomic modeling construction of human mandible though spiral CT scan, the anatomic model was changed into CAD model by Geomagic software. Then it was used to establish the triangular model where linear interpolation was implemented to yield smooth surfaces. The teeth were removed from the model as they made no difference to the mandible. 3D mandibular CAD model volume was 79812.04 mm^3^ and 21664.58 mm^2^. The reconstructed mandibular models were shown in Fig. 3.

### The internal microstructure of mandibular scaffold 3D mesh

**Figure 4:**
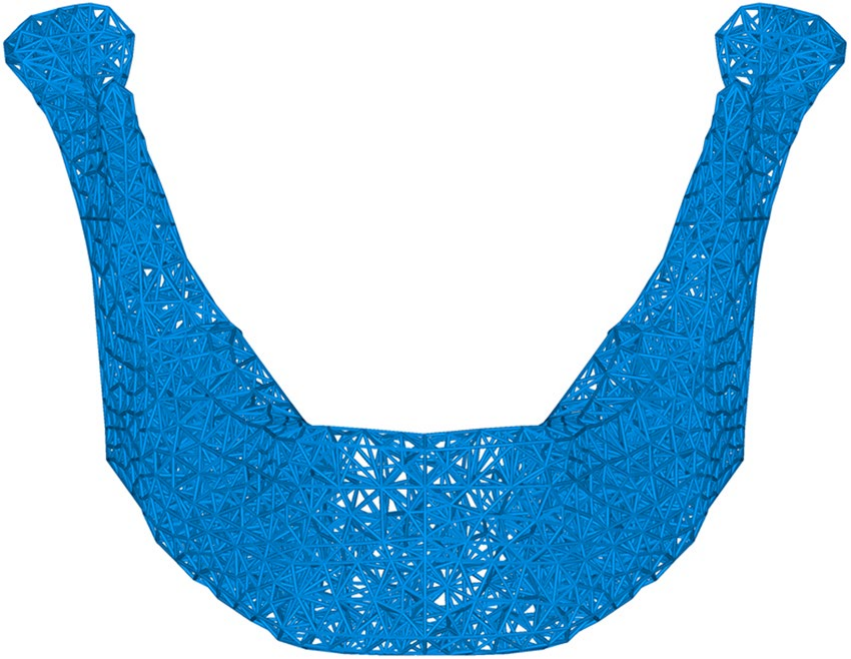
Design of mandibular scaffold 3D mesh internal microstructure.

The example given in this paper was a whole mandible defect case, and the reconstructed scaffold occupied all the mandible area. As shown in Fig. 4, meshing model was consisted of 8764 tetrahedrons elements in almost constant size, and scaffold model included 12316 beams with the same diameter of 0.7 mm. Once the scaffold model was determined, the whole finite element model of mandible with scaffold would be established subsequently. Meanwhile, the individual scaffold could be fabricated by 3D printing technology after smoothing procedure. Besides, there were many important factors in finite element analysis, such as 3D geometrical modeling, proper meshing, configuration of material characteristics, and so on. By properly simulating boundary condition and loading condition of chewing, the stress distribution on the mandible model was obtained. According to the uniformity principle of stress, the scaffold structure was optimized by bionic optimization method.

After optimization, the structure became more feasible. The strut’s section size could be reduced and scaffold structure was able to be better organized. The intact and edentulous mandible model volume was 79812.04 mm^3^ and scaffold model (Ti6Al4V) volume was 14864.45 mm^3^. Then the porosity (P_practical_) of the scaffold was calculated according to the following formula: P_practical_ = (V_prosthesis_−V_scaffold_)/V_prosthesis_ × 100% = (79812.04–14864.45)/79812.04 = 81.38%. Vprosthesis and V_scaffold_ standed for volume of mesh and bulk alloys, respectively.

### Preparation of Ti6Al4V scaffolds by EBM

**Figure 5:**
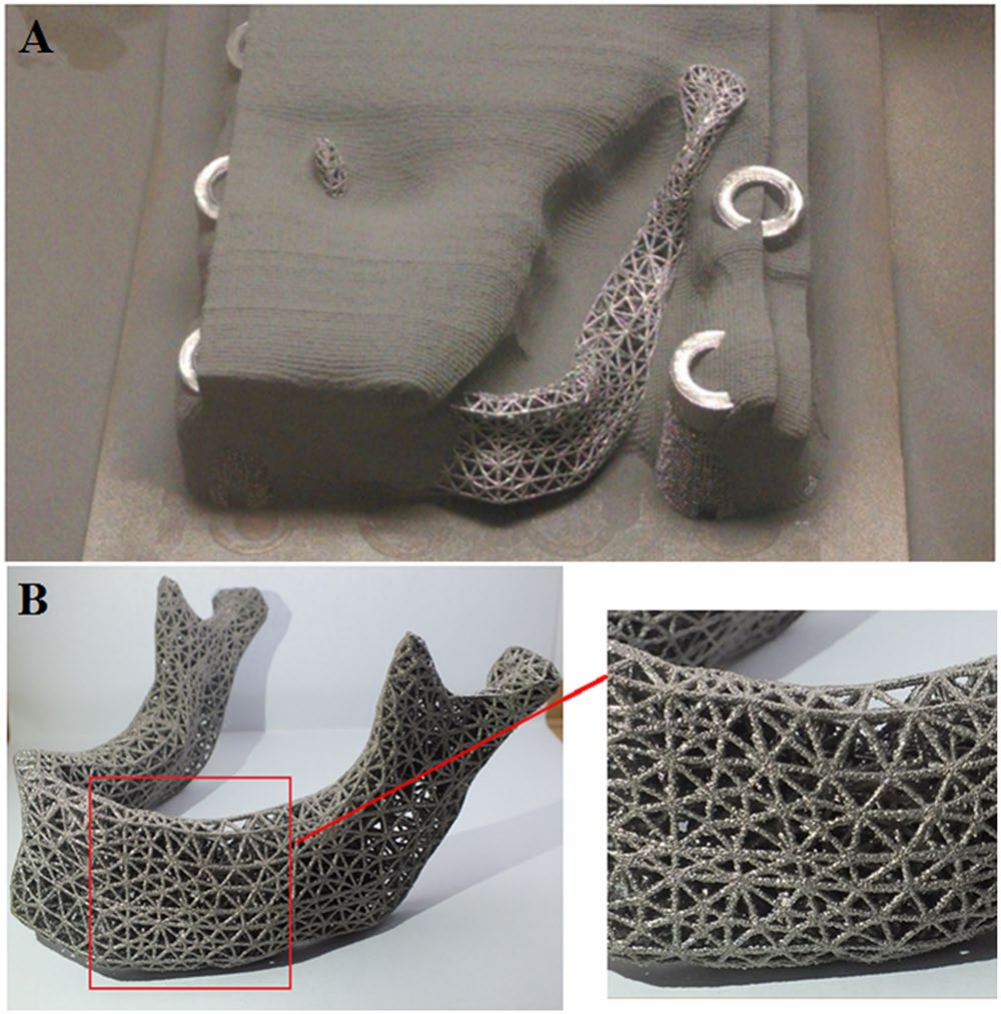
Preparation of Ti6Al4V scaffolds by EBM. (**A**) The parts are cleaned up to remove lose titanium powder lodged within the 3D mesh structure. (**B**) 3D mesh titanium mandibular prosthesis scaffold fabricate using EBM technology (Weight: 107 g; Porosity: 81.38%; Strut size: 0.7 mm).

The STL format file was inspected using Magics for repairing data process. Then these mesh structures were built layer-by-layer using the Ti-6Al-4V medical-grade powder with average diameter of 80 μm. A fabricated scaffold for mandible repair via EBM system was shown in Fig. 5A and the EBM mandibular scaffold samples were shown in Fig. 5B.

### Postoperative follow-up investigation

After the implantation, no numbness and repulsion were observed. The wound was healed well, and X-ray demonstrated that the prosthesis was fixed well without loosening and infection at postoperative 6 months. The facial features of the case were symmetrical, and the the masticatory and masticatory functions, as well as language function were restored.

### The process work-flow

**Figure 6:**
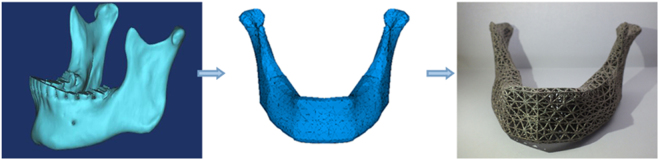
The process chain involved from image acquisition to production of a rapid prototype model composing three major steps: image acquisition, image post-processing and rapid prototyping.

Finally, we drew the process workflow: (1) acquisition of the CT data of the patients; (2) design with CAD and fabrication of custom EBM porous titanium implant; (3) implantation of the patient specific porous implant (Fig. 6).

### Animal models

#### The mandibular defects were repaired with titanium scaffolds mesh fabricated by EBM

After the implantation, all the animals had good physical and mental health. The wounds were healing well and no infection was detected. The transplant was stable in the animals and the mandibular continuity was restored.

**Figure 7:**
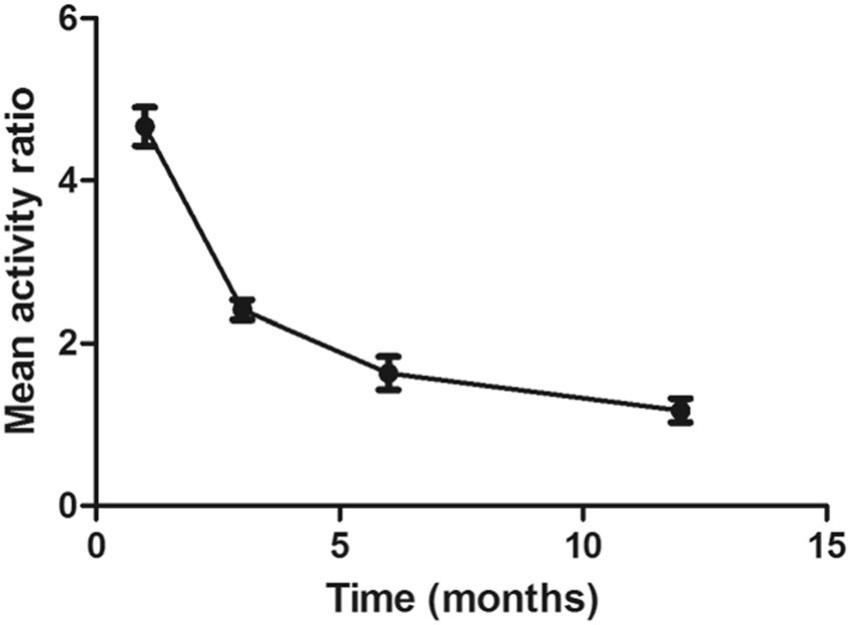
The ratio of radionuclide uptake between the grafted mandibular and the native normal mandibular. The highest ratio at the early postoperative stage suggested the rich blood supply and active bone metabolism. With the postoperative time prolonging, the ratio was nearly to 1.00, suggested that the grafted mandibular recovery well, and the blood supply and bone metabolism became stable.

Radionuclide bone imaging demonstrated that the ratio between the grafted mandible and the contralateral host mandible was highest at postoperative one month, and the ration presented declining trend during the subsequent period, until nearly to 1.0 (Fig. 7).

**Figure 8:**
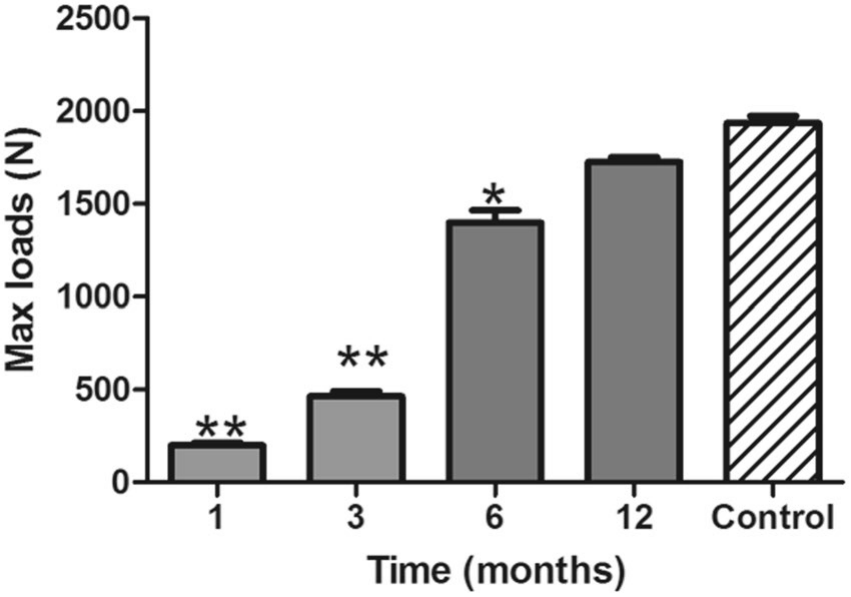
Mechanical testing for the grafted mandibular and native normal mandibular. After operation, the mechanical strength of the grafted mandibular was increased. At postoperative 12 months, the strength of the grafted mandibular was nearly to the normal native mandibular (*P* > 0.05). **Suggested *P* < 0.01; *suggested *P* < 0.05.

All the testing data were in accord with normal distribution, and parametric student’s t test was used for statistical analysis. The results for mechanical testing were shown in Fig. 8. From the figure, we could see that with the postoperative time prolonging, the strength of the grafted mandible was increased, and nearly to the normal mandible 12 months after operation.

**Figure 9:**
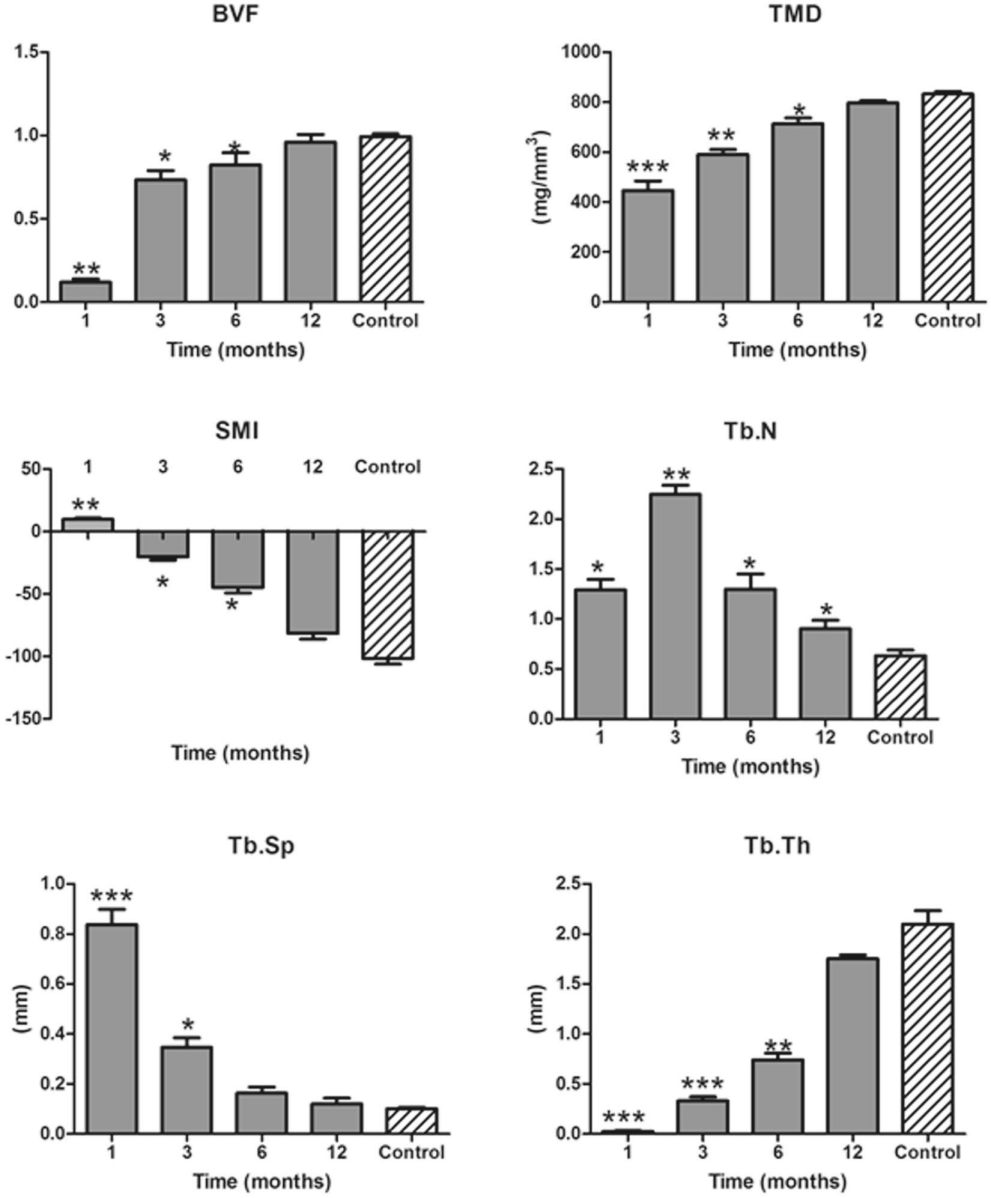
The data of micro-CT scan for the grafted and native mandibular. The data of BVF, TMD, and Tb.Th were increased, while the values of SMI and Tb.Sp exhibited decreased trend. All the parameters values were nearly to the normal native mandibular 12 months after operation. The data revealed that the grafted mandibular recovery normal. BVF: bone volume fraction; TMD: tissue mineral density; SMI: structure model index; Tb.N: trabecular number; Tb.Tn: trabecular thickness; Tb.Sp: trabecular separation. ***Suggested *P* < 0.001; **suggested *P* < 0.01; *suggested *P* < 0.05.

The data for micro-CT scan suggested that with the postoperative time prolonging, the values of BVF, TMD, and Tb.Th were increased, while the values of SMI and Tb.Sp exhibited down-regulated trend. Moreover, the values of the detecting indexes were nearly to the values of the normal mandible (Fig. 9).

## Discussion

The clinical goal of mandibular implants is to provide a substitute for muscles and loading, during either normal activities or trauma, which recreates the skeleton’s original stress-strain trajectories^5^. There is a need to fully stimulate the morphology and bio-mechanical properties of the mandibular bone, and use the tissue engineering to make a substitute for it. So it is necessary to explore new bionic design and manufacturing methods for mandibular prosthesis. A new technical solution for guiding surgery to repair mandibular defects is proposed, based on general popular tools in medical image processing including 3D model reconstruction, digital design, and fabrication via 3D printing^18,19^. 3D is printed in titanium with desirable strength, lightness, and bio-compatibility, therefore it’s possible to scan a patient and use the information to create a CAD file, and then print replacement joints and bones which are a perfect fit. Titanium is characterized by low density, high strength as well as corrosion resistant and bio-compatible ideal so as to be used in implant industries.

Several studies over the last decade have focused on CAD/CAM scaffold construction using various scaffold materials. In previous studies, the CAD programs were created by Materialise/Magics software for the reticulated mesh structures and CT scan for the stochastic foam structures. Hollister *et al*. proposed a simulation scheme for regularly repeating architectures and using constitutive equations relating^20^. However, the conditions of the mandible must be firstly known in order to do bionic design. Our study described a novel protocol that can be used to produce custom-made mesh scaffolds for mandibular bone regenerative medicine. Moreover, the scaffolds must be compatible with the anatomical defect and possess mechanical properties capable of bearing the loads encountered *in vivo*. It is a new approach, called tissue engineering (TE), that combines the advantages of titanium scaffold with internal 3D mesh structures, and eliminates problems such as donor site scarcity, immune rejection and pathogen transfer. However, the optimal 3D mesh structure is still not defined. Therefore, it is necessary to investigate the digital design and fabrication of these mesh scaffold to the utmost considering biological outcome^21,22^. Subsequently, the production of scaffolds with high controllability and repeatability in terms of mechanical and morphological parameters is strongly demanded. First, computerized tomography (CT) images are processed to reconstruct the 3D model of the mandible bone. Then the defect area is replaced by healthy contralateral bone to create the repaired model. With the repaired model as a reference object, the graft shape and cutline can be designed. Eventually, the physical model is fabricated via 3D printing.

CT is routinely used in current clinical practice to obtain a stack of two-dimensional (2D) slices of bone structures. As many biomechanical analyses and designs rely on the development of a reliable FEM or 3D model^15^, 3D surface reconstruction from these slices has been extensively studied. In this paper, the mandible was analyzed by three-dimensional finite element modeling, with regard to the bio-mechanical properties and stress distribution, which can provide a reference object for designing mandibular prosthesis. With sufficient communication between engineers and surgeons, an optimal porous structure can be designed via some common software platforms^23^. Murr *et al*. fabricated the Ti-6Al-4V cellular meshes and foams with numerous design elements by AM using EBM^12^. Heinl *et al*. has reported that cellular Ti6Al4V structures with interconnected macro porosity fabricated by EBM might have favorable long-term stability and were suitable for orthopedic applications^24^. Under such circumstance, designers can take advantages of the flexibility that is offered by 3D printing. Parts can be geometrically optimized for a high strength-to-weight ratio, designed to include functional components. Additionally, when it comes to orthopedic implants, custom is made to fit individual patients.

In our study, the rule of full stress is the most direct and effective method of the optimal design for shape truss. Combined with finite element theory, the general program is studied by the optimal theory of the rule of full stress. Through this program, the truss with different kinds and shapes in various load cases is designed automatically. After the FEA analysis of the network supporting structure, the structure is feasible. The upper and lower of supporting structure is coordinated, the frame beam section size is able to be reduced and mesh support in structure will be better fabricated. The structural mechanics of the mandibular truss implants are designed to distribute loads across the entire endplate and throughout the device. The truss implant designs have a distinctive open architecture, which accounts for up to 75% of the implant to be filled with graft material to maximize bone incorporation. Research in topological dimension theory led to the discovery of a novel geometry to create high-strength, lightweight web structures. 3D-printed technology utilizes engineering principles, such as structural mechanics and adjacent material reaction to produce innovative mandibular implants, that may actively participate in stimulating the healing process. A study carried out by Shan *et al*. demonstrated that maxillary and mandibular defects could be reconstructed by printed titanium meshes using computer-assisted surgery (CAS), moreover, no skin inflammation or titanium mesh exposure occurred in the follow-up^25^.

This study was carried out on the prosthesis structure optimization, guaranteed under the premise of strength, ease of construction redundant part, in the meanwhile the stress distribution was more uniform, bone graft material was more conducive to the growth of the stress environment and promoted the growth of bone graft material survival^26,27^. Some mechanical strength studies indicate the fabricated structures with 70% porosities satisfy the mechanical strength requirements needed for craniofacial applications^28^. We used 3D printing technology to print complete microfilament 0.7 mm diameter analog mandible defect bracket with the initial design proved feasible.

Although there is definitely a promising future for engineered grafts, their regular clinical application is still hard to implement successfully. Many studies assume that first of all a method was designed to fulfill the clinical effects; then the model would be refined considering a fixed prosthesis, dental implant area setting and other issues; finally optimization methods should be further improved. Nevertheless, everything has two sides, no exception to this study. For instance, the design included very narrow internal channels and excess material could get trapped during production, hence, it would become much more difficult to be removed. In addition, supports that some parts need hold them in place during the build process would also be difficult to be removed.

In this study, we also performed mandibular defect models to confirm the effects of titanium scaffolds mesh fabricated by EBM. After the implantation of titanium scaffolds mesh fabricated by EBM in the mandibular defect animal models, all the animals were healed well. Radionuclide bone imaging demonstrated that the blood supply was rich and bone metabolism was active at postoperative 1 months, suggesting the grafted mandibular recovered well, and no necrosis was observed. Micro-CT was performed to evaluate the remodeling and corticalization of the particle cancellous bone graft. The results demonstrated that the morphological parameters of the grafted mandibular were nearly to the normal native mandibular, suggesting the good recovery. All these results indicated the mandibular defects could be repaired with the titanium scaffolds mesh fabricated by EBM.

## Conclusions

In this article, our group’s present clinical protocol in which a innovative workflow was performed based on a complete CAD/CAE/CAM digital design plan combined the data from a computed tomography scan and fabricate 3D mesh Ti6Al4V scaffold. Using the novel software flow decreased the design time in comparison to other traditional CAD software flow. At the same time, the new EBM production process reduced the production time in comparison to conventional production processes. Besides, the titanium scaffolds mesh fabricated by EBM exhibited good biocompatibility. The option of designing a custom implant makes surgeons feel much easier to perform an operation, which results in reduction of the time needed in the surgery. The proposed methods have been adopted and their feasibility and validity have been verified.
